# Contribution of bone marrow derived cells to the pancreatic tumor microenvironment

**DOI:** 10.3389/fphys.2013.00056

**Published:** 2013-03-26

**Authors:** Christopher J. Scarlett

**Affiliations:** ^1^Food Bioactives and Pancreatic Cancer Biology Group, School of Environmental and Life Sciences, University of NewcastleOurimbah, NSW, Australia; ^2^Cancer Research Program, Garvan Institute of Medical ResearchDarlinghurst, Sydney, NSW, Australia

**Keywords:** bone marrow derived stem cells, pancreatic cancer, stroma, pancreatic stellate cells, fibrosis, immunosuppression, neovascularization

## Abstract

Pancreatic cancer is a complex, aggressive, and heterogeneous malignancy driven by the multifaceted interactions within the tumor microenvironment. While it is known that the tumor microenvironment accommodates many cell types, each playing a key role in tumorigenesis, the major source of these stromal cells is not well-understood. This review examines the contribution of bone marrow-derived cells (BMDC) to pancreatic carcinogenesis, with respect to their role in constituting the tumor microenvironment. In particular, their role in supporting fibrosis, immunosuppression, and neovascularization will be discussed.

## Introduction

It is long known that adult stem cells have remarkable flexibility in their differentiation repertoires. This plasticity allows adult stem cells, particularly those of bone marrow origin, to engraft alternative non-haematopoietic locations and differentiate into cell types appropriate to their new niche. This is particularly evident when the recipient organ is damaged (Poulsom et al., 2002; Alison et al., 2006). Bone marrow derived cells (BMDC) can either engraft or fuse to adopt, or be reprogrammed, to the differentiated state of the particular epithelia (Jang et al., 2004) [reviewed in (Herzog et al., 2003)]. This suggests that the endogenous stem cell of an organ is not confined to each specific organ but may be a dynamic system involving circulating BMDC with stem cell niche environments regulating recruitment, proliferation, and differentiation (Alison et al., 2006; Diaz-Flores et al., 2006). This may have significant implications concerning the evolution of cancers in many solid organs, including the pancreas.

Much debate has loomed as to the questionable functional significance of BMDC that have differentiated into epithelial cells of solid organs (Krause et al., 2001; Wagers et al., 2002; Bruscia et al., 2006), while very few studies have investigated this phenomenon in the pancreas (Wang et al., 2006; Marrache et al., 2008). Most studies have focused on restoring endocrine function following islet cell injury (Choi et al., 2003; Hess et al., 2003; Ianus et al., 2003; Lechner et al., 2004; Mathews et al., 2004; Taneera et al., 2006; Hasegawa et al., 2007; Gao et al., 2008b), with few studies assessing the contribution of BMDC to the exocrine pancreas, in particular in pancreatic cancer (Pan et al., 2009; Scarlett et al., 2011). Wang et al. (2006) describe the contribution of BMDC to pancreatic duct formation in neonatal mice, Marrache et al. (2008), and Watanabe et al. (2009) demonstrate that BMDC contribute to the pancreatic stellate cell (PSC) population in chronic pancreatitis, while Pan et al. (2009) and Scarlett et al. (2011) identified a contribution of BMDC to the PSC population in rat and mouse models, respectively, of chemical carcinogenesis of the pancreas.

**Figure 1:**
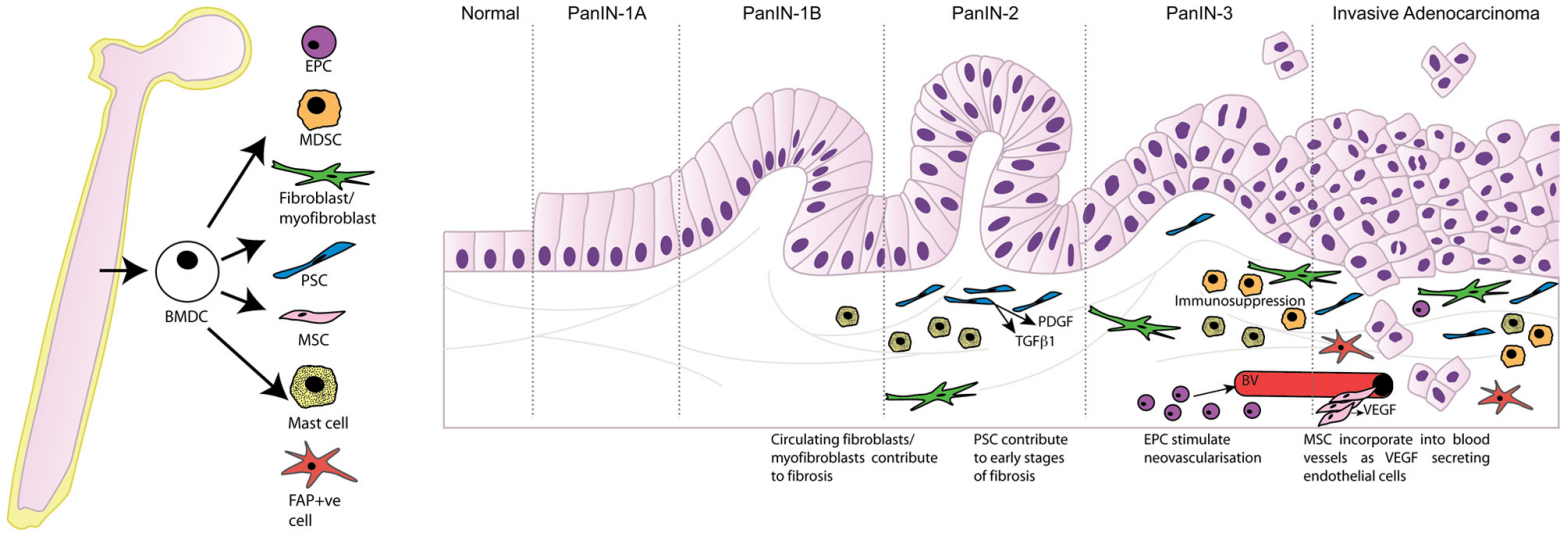
**Bone marrow derived cells contribute to the tumor microenvironment following recruitment to the desmoplastic stroma and differentiation into multiple cell types promoting tumor progression.** PanIN, pancreatic intraepithelial neoplasia; BMDC, bone marrow-derived cells; EPC, endothelial progenitor cells; MDSC, myeloid-derived suppressor cells; PSC, pancreatic stellate cells; MSC, mesenchymal stem cells; FAP, fibroblast activation protein-α; PDGF, platelet-derived growth factor; TGFβ1, transforming growth factor beta 1; VEGF, vascular endothelial growth factor; BV, blood vessel.

It is well-established that the pancreatic tumor microenvironment plays an integral role in driving pancreatic carcinogenesis and metastases, with the contribution of components of the pancreatic tumor microenvironment, such as PSC, myofibroblasts/fibroblasts, and inflammatory cells, reviewed elsewhere within this edition (Erkan et al., 2012; Evans and Costello, 2012). As such, this review will focus specifically on the contribution of BMDC in pancreatic carcinogenesis. In particular the significant role that BMDC has within the tumor microenvironment, and its implications for supporting pancreatic carcinogenesis, will be discussed. Table 1 and Figure 1 summarize the contribution of BMDC to the pancreatic tumor microenvironment.

**Table 1:** **Summary of bone marrow derived cell contribution to the pancreatic tumor microenvironment**.

**BMDC type**	**Contribution to pancreatic tumor microenvironment**	**References**
Myofibroblasts/fibroblasts	Contribution to myofibroblast/fibroblast populations within the pancreatic insulinoma, particularly at the tumor margin.	Direkze et al., 2004
	Circulating fibrocytes derived from BMDC and contributed to pancreatic fibrosis by differentiating into collagen-producing myofibroblasts.	Lin et al., 2012
Pancreatic stellate cells (PSC)	Contribution to early stages of fibrosis, and produced the growth factors PDGF and TGFβ1.	Akita et al., 2012
	Significant contribution to the activated PSC population, occurring early in pancreatic carcinogenesis. Express genes characteristic of peritumoral stellate cells compared to those not associated with malignancy.	Scarlett et al., 2011
Myeloid-derived suppressor cells (MDSC)	Immunosuppressive cell type within the stroma. Increased MDSC in the bone marrow, peripheral circulation, and tumor. Inhibition reduces intra-tumoral accumulation and prevents tumor growth.	Porembka et al., 2012
Mast cells	Immunosuppressive cell type within the stroma. Migration to the tumor microenvironment is an early event in carcinogenesis and is necessary for tumor growth.	Chang et al., 2011
Fibroblast activating protein (FAP) +ve cells	Immunosuppressive cell type within the stroma. Abrogation of FAP expression arrests pancreatic tumor growth.	Kraman et al., 2010
Pro-angiogenic cells for Neovascularization: Endothelial progenitor cells (EPC), Mesenchymal stem cells (MSC)	BM-derived endothelial progenitor cells (EPC) stimulate neovascularization and pancreatic cancer growth, via the CXCL5/CXCL8/CXCR2 axis. CXCR2 in particular was required for BM-derived EPC mobilization during pancreatic tumor growth.	Li et al., 2011
	BM-derived mesenchymal stem cells (MSC) contribute to neovascularization by migrating to fast growing tumors and incorporating into blood vessels as atypical VEGF-secreting endothelial cells.	Beckermann et al., 2008
	BM-derived pro-angiogenic cells are targets for Shh derived from the pancreatic tumors. Hh blockade disrupts tumor angiogenesis mediated through the impaired interaction BMDC with the neovasculature. Responsible for VEGF-independent neovascularization in pancreatic cancer.	Nakamura et al., 2010; Mizukami, 2012

*Only a representative selection of published data presented here*.

*BMDC, bone marrow-derived cells; PDGF, platelet-derived growth factor; TGFβ1, transforming growth factor beta 1; PSC, pancreatic stellate cells; MDSC, myeloid-derived suppressor cells; FAP, fibroblast activation protein-α; EPC, endothelial progenitor cells; CXCL5, epithelial neutrophil-activating peptide-78/chemokine; CXCL8, interleukin 8; CXCR2, interleukin 8 receptor β/chemokine receptor; MSC, mesenchymal stem cells; VEGF, vascular endothelial growth factor; Shh, sonic hedgehog; Hh, hedgehog*.

## Contribution of BMDC to epithelial tumor cells

A novel paradigm of epithelial cancer development was postulated following the early investigations of Houghton et al. (2004), where in a model of *Helicobacter felis* induced gastric carcinogenesis, the development of metaplasia and dysplasia was linked to the engraftment and expansion of the BMDC population, eventually giving rise to gastric adenocarcinoma (Houghton et al., 2004). It was thus hypothesized that in response to tissue damage, BMDC could migrate to and lodge in the stem/progenitor cell niche, expand as a clonal unit and repopulate the epithelium, and that mutations in these engrafted BMDC then lead to carcinogenesis [reviewed in (Alison et al., 2006)].

Studies of the exocrine pancreas have failed to provide evidence for differentiation of BMDC into epithelial tumor cells directly. Pan et al. (2009) demonstrated in a rat model of carcinogenesis that BMDC could modulate pancreatic cancer growth via incorporation into the microenvironment, specifically the PSC within the stroma (Pan et al., 2009). These findings were strengthened in a more recent study by Scarlett et al. (2011), who also demonstrated a significant contribution of BMDC to the peritumoral stroma in a mouse model of pancreatic carcinogenesis (Scarlett et al., 2011). These data suggest that while BMDC do not contribute directly to pancreatic epithelial tumorigenesis, they play a significant and supportive role in promoting carcinogenesis via interactions within the tumor microenvironment. This will be discussed in more detail below.

## BMDC and the tumor microenvironment

There is increasing evidence that the tumor microenvironment influences tumor proliferation and survival, metastasis, resistance to therapy and escape from immune control [reviewed in (Feig et al., 2012)]. The desmoplastic stroma associated with pancreatic cancer is made up of a heterogeneous population of cells including immune cells, stellate cells, blood vessels, extracellular matrix (ECM), fibroblasts, and myofibroblasts. However, the major source of pancreatic stromal cells is not well-understood. While conversion of resident cells within normal tissue stroma are obvious candidates, increasing evidence is emerging that BMDCs are a source of non-resident stromal cells that contribute significantly to the stroma, and thus aid in the progression and invasion of pancreatic malignancy (Luo et al., 2012).

### Contribution of BMDC to fibrosis

The role of myofibroblasts in fibrosis is well-documented in numerous solid tumors (Direkze et al., 2004), and along with fibroblasts and ECM proteins they produce, are key components of the desmoplastic response to tumors (De Wever and Mareel, 2003). Early studies by Direkze et al. (2004) demonstrated in a mouse model of pancreatic insulinoma that BMDC contribute to both myofibroblast and fibroblast populations within the tumors, particularly at the tumor margin. This study raised many questions, and suggested that the development of the pancreatic tumor stroma may be a less localized phenomenon than first thought (Direkze et al., 2004).

More recently, Lin et al. (2012) demonstrated in a mouse model of caerulein-induced pancreatitis that circulating fibrocytes were indeed derived from BMDC. These fibrocytes could engraft to the pancreas from peripheral circulation, and contributed to pancreatic fibrosis in part by differentiating into collagen-producing myofibroblasts. Further, when genetically modifying the BMDC, the severity of the fibrosis within the pancreas could be altered, suggesting that BMDC can function as fibrogenic cells (Lin et al., 2012).

### Contribution of BMDC to the pancreatic stellate cell population

PSC are resident myofibroblast-like cells existing in the periacinar space of the exocrine pancreas. In a healthy pancreas, PSC (quiescent) comprise approximately 4% of pancreatic cells, and exist in a periacinar dissemination. There is increasing evidence to suggest that PSC are key participants in the pathogenesis of pancreatic exocrine diseases, particularly in the production of the abundant fibrous stroma, which is a feature of pancreatic cancer (Apte et al., 1998, 2004; Bachem et al., 1998; Hwang et al., 2008; Vonlaufen et al., 2008; Phillips, 2012).

Akita et al. (2012) demonstrated in a rat model of pancreatic fibrosis that bone marrow derived activated PSC contribute to the early stages of fibrosis, and produced the growth factors PDGF and TGFβ1, key growth factors involved in the cross-talk between pancreatic tumor cells and PSC that contribute to tumor invasion and metastasis (Vonlaufen et al., 2008; Akita et al., 2012).

In a recent study investigating the potential role for BMDC in pancreatic injury, regeneration and carcinogenesis, Scarlett et al. (2011) observed that there was significant BMDC recruitment to the inflammatory infiltrate at the time of pancreatic injury (caerulein-induced chronic pancreatitis) consistent with previous reports (Minami et al., 2005; Sparmann et al., 2010; Scarlett et al., 2011), which was transient as cell numbers diminished over time to low levels when exocrine regeneration was complete. Interestingly, PSC remained amongst the residual population of BMDC, suggesting that BMDC play a role in supporting the regenerative process, but do not transform to contribute to the regenerative epithelium itself. As observed for chronic pancreatitis, increased recruitment of BMDC to the pancreas occurred following DMBA-induced carcinogenesis, which predominantly included inflammatory infiltrate, while activated PSC were also present (Scarlett et al., 2011). In an earlier study, Erkan et al. (2010) used transcript profiling to identify markers to differentiate PSC associated with chronic pancreatitis against those of pancreatic cancer, with the ultimate aim of subtyping PSC into either inflammation- or tumor-associated-PSC. Erkan et al. identified Pre-B-cell leukemia transcription factor 1 (PBX1) to be upregulated in inflammation-associated PSC compared to tumor-associated PSC, while cadherin EGF LAG seven-pass G-type receptor 3 (CELSR3) expression was upregulated in tumor-associated PSC compared to that of inflammation-associated PSC (Erkan et al., 2010). In the study of Scarlett et al. (2011), expression of CELSR3 in tumor-associated BMDC suggests that there was modification of these PSC by the tumor microenvironment, and that tumor associated BMDC PSC may be retained in the peri-tumoral stroma, whilst those associated with pancreatitis are not. This is supported by studies where bone marrow-derived mesenchymal stem cells (MSC) preferentially localize to regions of pancreatic tumor growth (Kallifatidis et al., 2008) and have been shown to transform into tumor-associated myofibroblasts in insulinomas (Direkze et al., 2004).

While most tumor associated activated PSC are thought to arise from endogenous quiescent PSC, evidence now exists that a proportion of these are bone marrow derived, and display different phenotypes depending on whether they are recruited to an inflammatory or a carcinogenic pancreas. In pancreatic carcinogenesis, and in chronic pancreatitis, BMDC contribute significantly to the activated PSC population, with evidence suggesting that this process occurs as an early event in pancreatic carcinogenesis (Scarlett et al., 2011). Those associated with pancreatic cancer express genes characteristic of peritumoral stellate cells as compared to those not associated with malignancy, providing further evidence that BMDC play an important role in supporting pancreatic carcinogenesis, the mechanisms of which remain to be elucidated.

Of interest are the mechanisms by which BMDC are recruited to the stroma. Are they recruited by resident PSC themselves? Or do they migrate to regions of fibrosis via inflammatory signaling? Or both? Within the early stages of pancreatic injury, resident quiescent PSC become activated and promote fibrosis via the expression of ECM proteins, cytokines and growth factors such as PDGF and TGFβ1. These growth factors are known activators of PSC and in an autocrine manner leads to persistent PSC activation, promoting further fibrosis. A consequence of fibrosis is the increased recruitment of BMDC to the stroma, including BM derived PSC (Akita et al., 2012). This suggests a role for tissue repair, however, depending on the signals that the migrating BMDC receive (such as those observed in a tumor-associated microenvironment), this may lead to an environment supporting tumor progression as opposed to tissue repair, as described above (Erkan et al., 2010) and [reviewed in (Evans and Costello, 2012)]. Clearly there is more complexity involved than just an inflammation, PSC activation, BMDC recruitment and differentiation loop occurring in the fibrotic microenvironment. Delineating the exact mechanisms by which this occurs will lead to novel therapeutic strategies targeting the fibrotic stroma.

### Contribution of BMDC to immunosuppression

It is long known that inflammation is crucially linked to pancreatic cancer development, as evidenced by chronic pancreatitis being a major risk factor (Lowenfels et al., 1993). The mechanisms by which inflammation exacerbates an individual's risk of developing pancreatic cancer are yet to be fully understood. It is becoming increasingly evident that immune cells make up approximately 50% of the tumor mass (tumor and stroma) (Clark et al., 2007; Scarlett et al., 2011), with immunosuppressive cells, such as Myeloid-derived suppressor cells (MDSC) and mast cells, predominant.

#### Myeloid-derived suppressor cells (MDSC)

*MDSC* are a heterogeneous population of undifferentiated and immature myeloid immunosuppressive cells. Tumor-induced alterations in bone-marrow myelopoiesis are driven by growth factors and cytokines secreted by the tumor, which leads to expansion and mobilization of MDSC. Once mobilized to the stroma, MDSC promotes tumor growth, invasion, and tumor-induced immunosuppression and host immune evasion by inhibiting lymphocyte activation and antigen recognition (Serafini et al., 2006; Marigo et al., 2008; Gabrilovich and Nagaraj, 2009). Of importance, Porembka et al. (2012) recently demonstrated that patients with pancreatic adenocarcinoma exhibited increased MDSC in the bone marrow and peripheral circulation, as well as within the tumor itself, while inhibition of MDSC with the aminobisphosphonate zoledronic acid, reduces intra-tumoral accumulation of MDSC preventing tumor growth and increasing T-cell recruitment (Porembka et al., 2012).

#### Mast cells

*Mast cells* regulate adaptive immune responses via the release of cytokines and other immunomodulatory factors, which promote immune suppression and potentially tumor progression (Galli et al., 2005; Kalesnikoff and Galli, 2008). Chang et al. (2011) demonstrated that migration of bone-marrow derived mast cells to the tumor microenvironment is an early event in carcinogenesis and is necessary for tumor growth, the mechanisms of which remain to be elucidated (Chang et al., 2011).

The importance of an additional immunosuppressive cell type within the stroma was further defined through a recent study by Kraman et al. (2010), who demonstrated that a sub-population of stromal cells that express fibroblast activation protein (FAP) suppress the immune response and that abrogation of FAP expression arrests the growth of pancreatic tumors, potentially by removing their inhibitory effect on the host's immune response. (Kraman et al., 2010; Schreiber and Rowley, 2010). Thus, novel immunostimulatory therapeutic strategies targeting these immunosuppressive mechanisms may prove effective against pancreatic adenocarcinomas.

### Contribution of BMDC to neovascularization

A consequence of the excessive desmoplastic stroma is a profound alteration of the tumor vasculature, with the subsequent vascular dysfunction presenting a significant barrier to the delivery of chemotherapeutic agents to the tumor cells. As such, strategies targeting elements within the stroma have become more extensively studied (Olive et al., 2009; Provenzano et al., 2012). The mysteries surrounding hypovascularity in pancreatic cancer have been reviewed elsewhere (Feig et al., 2012), with many questions remaining unanswered at present. This section will focus solely on the contribution of BMDC to tumor neovascularization, and the implications therein.

Most solid tumors require a vascular supply to provide oxygen and nutrients to enhance tumor progression and invasion. The tumor can either exploit existing vessels, or recruit and mobilize BMDC to induce neovascularization. BMDC are increasingly valued to be important contributors to the expansion of the tumor vasculature, however, the mechanisms of BMDC recruitment and mobilization by the tumors are yet to be fully understood (Lyden et al., 2001; Shojaei et al., 2007; Chan et al., 2009).

Endothelial progenitor cells (EPCs) originate from BMDC and possess the capacity to differentiate into mature endothelial cells, contributing to the complex process of tumor neovascularization. There is evidence that tumor angiogenesis can be stimulated by tumor cell secreted CXC chemokine ligands CXCL5 and CXCL8, via their common receptor CXCR2 (Wente et al., 2006; Raman et al., 2007). Li et al. (2011) recently demonstrated a role for BM-derived EPCs in stimulating neovascularization and pancreatic cancer growth, with EPCs mobilized by the pancreatic cancer cells, and that targeting the CXCL5/CXCL8/CXCR2 axis impaired EPC mobilization, proliferation, differentiation and neovascularization. CXCR2 in particular was required for EPC mobilization during pancreatic tumor growth, indicating a critical role for CXCR2 in the regulation of BM-derived progenitor cells with respect to tumor angiogenesis (Li et al., 2011).

BM-derived MSC have also been shown to migrate toward tumor hypoxia-induced secretion of growth factors (VEGF, PDGF, EGF) in pancreatic tumors, and contribute to neovascularization by homing to fast growing tumors and incorporating into blood vessels as atypical VEGF-secreting endothelial cells (Beckermann et al., 2008). For pancreatic cancer in particular, anti-vascular therapies have failed to show a survival benefit, most likely due to the heterogeneous tumor microenvironment that creates a hypovascular milieu. It is clear that the tumor microenvironment plays a critical role in pancreatic tumor development and progression and that circulating BMDC have an inherent capacity to differentiate into endothelial cells to form new vasculature (Gao et al., 2008a). Recently, Nakamura et al. (2010) identified that BM-derived pro-angiogenic cells are potential targets for Sonic Hedgehog (Shh), derived from the pancreatic tumors, and that Hh blockade can disrupt tumor angiogenesis *in vivo*, which is mediated through the impaired interaction BMDC with the neovasculature in pancreatic cancer (Nakamura et al., 2010). As such, it was speculated that induction of angiogenesis via Shh may mediate VEGF-independent neovascularization in pancreatic cancer, thereby serving as a potential mechanism for resistance to anti-VEGF therapy (Mizukami, 2012).

There is mounting evidence that BMDC contribute to the neovascularization in pancreatic cancer, however, pancreatic cancer remains a hypovascular tumor with significant perfusion impairment. With a distinct lack of efficacy of anti-angiogenic therapies for pancreatic cancer [reviewed in (Feig et al., 2012)], alternative approaches targeting the pancreatic cancer vasculature are required.

## Concluding remarks

In conclusion, pancreatic carcinogenesis is an extremely aggressive, and complex malignancy with very few effective therapeutic modalities. Pancreatic cancer is characterized by an extensive desmoplastic stroma that interacts with the tumor cells, where together they drive tumor progression, invasion and metastasis. In addition, the stroma provides a physical barrier, denying the tumor cells access to chemotherapeutic agents. Future research priorities should be focused on further understanding the complex tumor-stroma relationships, which is key for facilitating the development of potential novel therapeutic strategies. Such strategies may include reducing tumor neovascularization by targeting the ablation of BM derived EPC (Li et al., 2011); or genetic manipulation of (1) BMDC to block Hh signaling and regress the tumor vasculature (Nakamura et al., 2010), or (2) BM derived PSC by using the *hGFAP* promotor to develop targeted gene/drug therapy (Ding et al., 2009); or by unmasking the host's immune response by targeting BMD stromal fibroblasts that contribute to immunosuppression and tumor progression (Kraman et al., 2010). This review discussed the role of BMDC in pancreatic carcinogenesis, in particular their contribution to the tumor microenvironment, where they play a significant role in fibrosis, immunosuppression and neovascularization, and may provide opportunities for novel therapeutic strategies for pancreatic cancer.

### Conflict of interest statement

The author declares that the research was conducted in the absence of any commercial or financial relationships that could be construed as a potential conflict of interest.
